# Protocol for Evaluating *In Vivo* the Activation of the P2RX7 Immunomodulator

**DOI:** 10.1186/s12575-022-00188-6

**Published:** 2023-01-04

**Authors:** Serena Janho dit Hreich, Thierry Juhel, Paul Hofman, Valérie Vouret-Craviari

**Affiliations:** 1 Université Côte d’Azur, CNRS, INSERM, IRCAN, 28 avenue de Valombrose, 06108 Nice, France; 2 FHU OncoAge, Pasteur Hospital, 30 voie Romaine, 06001 Nice, France; 3 Laboratory of Clinical and Experimental Pathology and Biobank, Pasteur Hospital, 30 voie Romaine, 06001 Nice, France; 4 Hospital-Related Biobank (BB-0033-00025), Pasteur Hospital, 30 voie Romaine, 06001 Nice, France

**Keywords:** P2RX7, P2X7 receptor, Purinergic receptors, ATP, Macropore, TOPRO-3, Activity

## Abstract

**Background:**

P2RX7 is a purinergic receptor with pleiotropic activities that is activated by high levels of extracellular ATP that are found in inflamed tissues. P2RX7 has immunomodulatory and anti-tumor proprieties and is therefore a therapeutic target for various diseases. Several compounds are developed to either inhibit or enhance its activation. However, studying their effect on P2RX7’s activities is limited to in vitro and ex vivo studies that require the use of unphysiological media that could affect its activation. Up to now, the only way to assess the activity of P2RX7 modulators on the receptor *in vivo* was in an indirect manner.

**Results:**

We successfully developed a protocol allowing the detection of P2RX7 activation *in vivo* in lungs of mice, by taking advantage of its unique macropore formation ability. The protocol is based on intranasal delivery of TO-PRO™-3, a non-permeant DNA intercalating dye, and fluorescence measurement by flow cytometry. We show that ATP enhances TO-PRO™-3 fluorescence mainly in lung immune cells of mice in a P2RX7-dependant manner.

**Conclusions:**

The described approach has allowed the successful analysis of P2RX7 activity directly in the lungs of WT and transgenic C57BL6 mice. The provided detailed guidelines and recommendations will support the use of this protocol to study the potency of pharmacologic or biologic compounds targeting P2RX7.

**Supplementary Information:**

The online version contains supplementary material available at 10.1186/s12575-022-00188-6.

## Background

The purinergic P2X family of receptors is a family of 7 receptors that are all activated by extracellular ATP (eATP) with various affinities. Unlike the other 6 receptors, P2RX7 requires high levels of eATP (in the range of hundreds of micromolar) to be activated. Such levels of eATP are found in inflamed tissues such as the tumor microenvironment or fibrotic sites [1].

P2RX7 is a receptor with pleiotropic activities. Its activation leads to calcium influx and potassium efflux, a feature it shares with all members of the P2X family and that has been shown to trigger activation of various signaling pathways [2]. However, P2RX7 has its own particularities: its activation also leads to a macropore opening at the plasma membrane that allows the non-selective entry of macromolecules (up to 900 Da) to the cell. Compromising membrane integrity through macropore opening by a prolonged or sustained activation (over 1 hour) of P2RX7 could also lead to cell death [3]. Moreover, activation of P2RX7 leads subsequently to the assembly of the NLRP3 inflammasome and the release of mature IL-1β and IL-18 from the cell [4].

Due to its ability to induce cell proliferation and death but also to modulate the immune response, several approaches are undertaken to either inhibit or enhance its activation, by engineering chemical molecules [5, 6] or nanobodies [7] targeting the receptor. Three assays are classically and routinely used to evaluate their ability to modulate P2RX7’s activation, based on the 3 hallmarks of P2RX7:

- Calcium influx: the goal is to measure intracellular calcium concentrations using cell permeant fluorescent calcium indicators such as Fluo-4 AM or Fura 2. Calcium concentration could be assessed by spectrophotometry, microscopy or flow cytometry. Calcium influx could also be detected by measuring ion fluxes by electrophysiology techniques such as patch-clamp.
- Macropore opening: To evaluate the opening of the macropores, non-permeant DNA intercalating fluorescent dyes of high molecular weight (up to 900 Da) are used, such as TO-PRO™-3 or Ethidium bromide. Fluorescence is assessed by spectrophotometry, microscopy or flow cytometry.
- NLRP3 inflammasome activation is determined by checking adaptor protein ASC oligomerization, caspase-1 cleavage or IL-1β/IL-18 release. It is assessed by immunofluorescence, western blot, flow cytometry or ELISA.

However, these assays are in vitro or ex vivo-based and rely on using unphysiological medias that could interfere with P2RX7 activation. Indeed, the ionic composition of media is crucial. ATP-induced cell death has been shown to be delayed in low-salt medias [8]. Moreover, macropore formation is impacted by Na^+^, iodide, thiocyanate and chloride-enriched medias, since increasing concentration of these ions inhibited ethidium uptake by cells [9]. Channel opening is also affected by external anions such as glutamate that potentiates human P2RX7 activation whereas chloride and iodide decreased it [10]. Since P2RX7 activation hangs in the balance of ions concentrations in media, measurement of its activation in vitro and its translation *in vivo* can be not accurate. For example, under pathological conditions, glutamate levels increase in the extracellular space during nerve injury, that could affect P2RX7 activation [11].

Since compounds targeting P2RX7 are tested in vitro, little is known on their impact on P2RX7 activities *in vivo*, i.e. in complete physiological conditions. Up to now, *in vivo* P2RX7 activation state is only seen indirectly through eATP levels or IL-1β/IL-18 production. Therefore, we aimed at setting up a way to measure its activation *in vivo* and developed a protocol based on intranasal delivery of TO-PRO™-3 and fluorescence measurement by flow cytometry.

## Results

### General Strategy to Measure P2RX7 Activity *In Vivo*

**Fig. 1:**
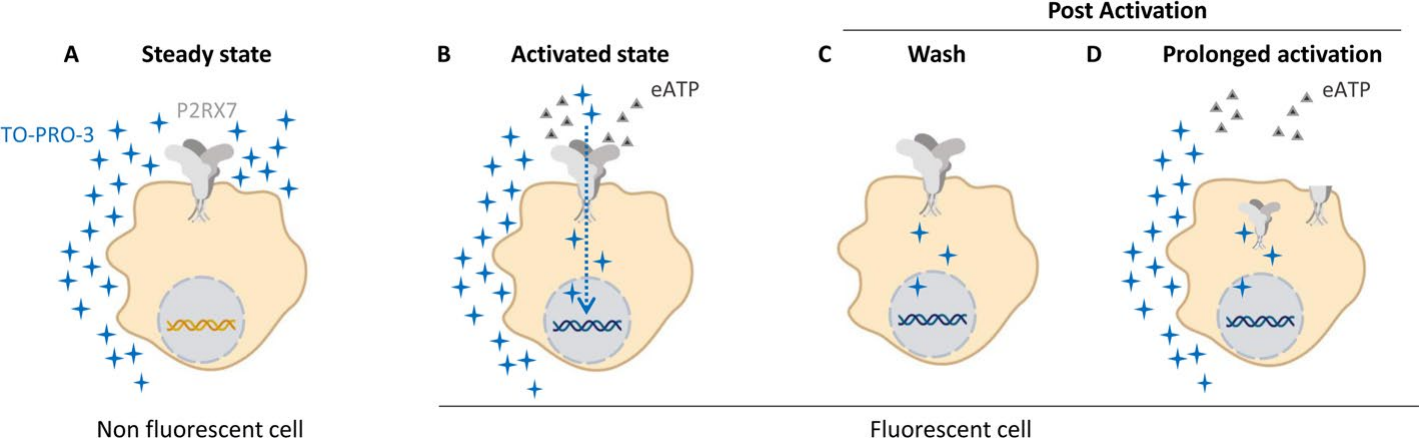
Activation of P2RX7 and macropore opening using TO-PRO™-3 dye

Since calcium influx is a common feature of P2X receptors and that NLRP3 assembly is a step downstream of P2RX7 but also not specific to P2RX7, we opted to use macropore opening, which directly represents the activation state of the receptor, as a readout for P2RX7 activity (Fig. 1). Several dyes are available for measuring macropore opening. We opted to use the TO-PRO™-3 dye (Invitrogen) since it is the most sensitive non-permeant DNA intercalating dye and was shown to work best in flow cytometry [12].

When the cell is in steady state (absence of eATP, membrane integrity) TO-PRO™-3 does not enter the cell and the cell remains non fluorescent (Fig. 1A). In the presence of eATP, P2RX7 is activated and macropores are open, permitting the entry of TO-PRO™-3 into the cell. TO-PRO™-3 binds to DNA and renders the cell fluorescent (Fig. 1B). Macropore formation is a reversible mechanism. However, once P2RX7 is activated, the cell remains fluorescent since TO-PRO™-3 has already bound to DNA, even if no eATP remains or if P2RX7 is internalized [13, 14] or cleaved [15] during a prolonged activation (Fig. 1C and D), allowing the detection of P2RX7 activation at any point in time.

Channel opening and P2RX7-dependent calcium influx are detected few seconds after ATP stimulation. However, detection of macropore formation is much slower due to the incorporation of fluorescent dyes in DNA. and can take up to 1 hour to detect all activated receptors [12]. We have previously shown in a time course experiment of ATP-induced TO-PRO™-3 uptake that a range between 20 and 30 minutes post stimulation is the ideal time point at which the TO-PRO™-3 signal is well above background [5]. Moreover, since prolonged activation of P2RX7 (over 1 hour) can also lead to cell death in a macropore-dependent manner and since TO-PRO™-3 can also detect dead cells, it is crucial to add a Live/Dead marker. Indeed, excluding dead cells to reduce background noise and macropore-unrelated TO-PRO™-3 staining is important for accurate P2RX7-activation measurement. Therefore, to detect P2RX7-related TO-PRO™-3 fluorescence and to rule out detection of ATP-induced cell death, we chose to activate P2RX7 for 30 minutes *in vivo*.

### ATP Activates P2RX7 in the Lung and Increases *In Vivo* TO-PRO™-3 Fluorescence

**Fig. 2:**
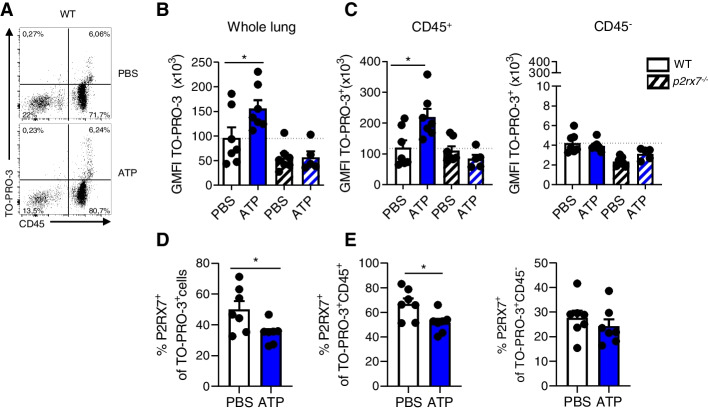
P2RX7 is activated *in vivo.* **A** Dotplot gated on live single cells of WT mice whole lungs. **B** GMFI of TO-PRO™-3^+^ cells in whole lung of WT and *p2rx7*^*−/−*^ mice. **C** GMFI of TO-PRO™-3^+^ cells in CD45^+^ cells (left) and CD45^−^ cells (right) in WT and *p2rx7*^*−/−*^ mice. **D** Percentage of P2RX7^+^ cells among TO-PRO™-3^+^ cells, **E** Percentage of P2RX7^+^ cells among TO-PRO™^-^3^+^CD45^+^ cells (left) and TO-PRO™-3^+^CD45^−^ cells (right). One point represents one mouse, data is represented as mean ± SEM. Two-tailed unpaired *t*-test. *p < 0.05. WT: Wildtype, GMFI: geomean fluorescence intensity

We first analyzed TO-PRO™-3 fluorescence in the whole lung. We observed an increase of fluorescence in WT mice receiving ATP compared to PBS (Fig. 2A and B), indicative of macropore opening and therefore activation of P2RX7. In order to know if this increase is specific of P2RX7, the experiment was also done on *p2rx7*^*−/−*^ mice, where the increase of TO-PRO™-3 fluorescence in the ATP group was not observed and was similar to WT-PBS mice (Fig. 2B). No difference was observed in the percentage of TO-PRO™-3 positive cells (supp Fig. 1A-C). These results suggest that TO-PRO™-3 fluorescence after ATP administration was specific to P2RX7 and indicative of its activation state.

To go further, we analyzed TO-PRO™-3 fluorescence in immune and non-immune cells. We show that WT-ATP mice exhibit an enhanced fluorescence compared to WT-PBS mice only in immune cells (Fig. 2C), and that was only observed in WT mice, demonstrating furthermore the requirement of P2RX7 for the TO-PRO™-3 signal observed.

We also show that TO-PRO™-3^+^ cells express P2RX7. However, percentage of P2RX7^+^ cells among TO-PRO™-3^+^ cells was decreased in ATP-mice (Fig. 2D and E), but not in TO-PRO™-3^+^CD45^−^ cells (Fig. 2E). This is not surprising since it has been shown that sustained activation of P2RX7 leads to the internalization of the receptor [14] or its cleavage [15] after its activation, explaining the possibility of TO-PRO™-3^+^P2RX7^−^ cells.

### P2RX7 Is Mainly Expressed and Activated in Lung Immune Cells

**Fig. 3:**
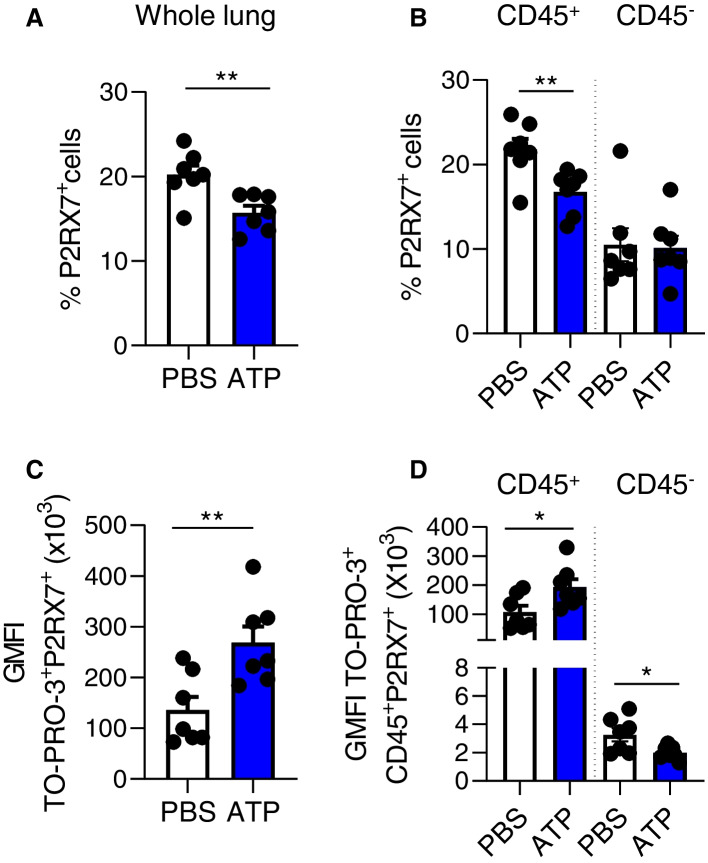
TO-PRO™-3 fluorescence is increased in P2RX7^+^ cells in WT mice. **A** Percentage of P2RX7^+^ cells in the whole lung of WT mice. **B** Percentage of P2RX7^+^ cells in CD45^+^ and CD45^−^ cells. **C** GMFI of TO-PRO™-3 in P2RX7^+^ cells. **D** GMFI of TO-PRO™-3 in P2RX7^+^CD45^+^ and P2RX7^+^CD45^−^ cells. One point represents one mouse, data is represented as mean ± SEM. Two-tailed unpaired *t*-test. *p < 0.05, **p < 0.01. WT: Wildtype, GMFI: geomean fluorescence intensity

Since we observed a decrease of P2RX7 expression in TO-PRO-3^+^ cells (Fig. 2D and E), we investigated furthermore its expression in whole lungs of mice. We show that the expression of P2RX7 was decreased in all cells (Fig. 3A) but also in immune cells (Fig. 3B), supporting furthermore the idea of an internalization of P2RX7 after its activation. Moreover, we show that P2RX7 is more expressed by immune cells than non-immune cells (Fig. 3B). Even though WT-ATP mice show a decrease of P2RX7 expression, P2RX7^+^ cells show a higher TO-PRO™-3 fluorescence in all cells (Fig. 3C) and in immune cells (Fig. 3D), meaning that P2RX7^+^ cells are indeed activated by ATP.

Since TO-PRO™-3 fluorescence (Fig. 2C) and P2RX7 expression (Figs. 2E and 3B) were unchanged after ATP delivery in CD45^−^ cells, we can speculate that P2RX7-expressing immune cells are the main cells activated by ATP in the lung. However, we observed a decrease of TO-PRO™-3 fluorescence in CD45^−^ P2RX7^+^ cells (Fig. 3D).

## Discussion

This protocol was set up to assess P2RX7 activation *in vivo*, by taking advantage of its property of macropore formation. Among P2X receptors, a permeability for large cations was also described for P2RX2 and P2RX4. However, P2RX7 is activated by high concentrations of eATP (0.5 to 1 mM), whereas P2RX2 and P2RX4 are activated by much lower concentrations (3 to 10 μM). Unlike P2RX7, P2RX2 and P2RX4 receptors desensitize after ATP stimulation. Even though P2RX4 is broadly expressed in most tissues of the body, it is preferentially localized in lysosomes where the acidity prevents its activation [16**]**. Based on these properties, we hypothesized that the macropore activity of P2RX2 and P2RX4 will not interfere with our assay. This is indeed the case since no increase in TO-PRO**™**-3 signal was observed in *p2rx7*^*−/−*^ mice (Fig. 2B).

Our protocol allows the measurement of P2RX7 activation in lungs and is based on TO-PRO™-3 uptake by cells and detection by flow cytometry. Flow cytometry was previously shown as a sensitive tool to study P2RX7 activation in vitro [12] and is also efficient for analysis of an *in vivo* activation of the receptor as shown in this study. Moreover, flow cytometry allows the reduction of background noise by excluding non P2RX7-dependent TO-PRO™-3 staining of dead cells. The other advantage of using flow cytometry is coupling TO-PRO™-3 with other surface markers to distinguish P2RX7 activation at the single cell level. We only studied its activation in immune versus non-immune cells, however, one can easily increase the number of markers during the surface staining step to precisely identify the subtypes of immune and non-immune cells activated by ATP.

To our knowledge, we show for the first time in a direct manner that P2RX7 is mainly activated in lung immune cells of mice, as per an increase of TO-PRO™-3 fluorescence. P2RX7 has a pro-inflammatory role since it releases IL-1β and is at the etiology of inflammatory diseases. Nonetheless, direct activation of the receptor i.e. calcium influx or macropore formation was never assessed *in vivo*. However, expression of P2RX7 on hematopoietic cells [17, 18], ATP levels [19, 20] and IL-1β release [21, 22] were shown *in vivo* to be important for airway inflammation in mouse models. Even so, these experiments require the use of transgenic and chimeric mice and do not evaluate the receptor activation status. Our protocol could be used instead for accurate and easier assessment of the activation of P2RX7. We previously showed using human tumor lung adenocarcinoma (LUAD) tissues that P2RX7 is expressed in tumor associated immune cells whereas non-immune cells do not express P2RX7. Further, we showed that the overall activity of P2RX7 is impaired by expression of the C-terminal domain truncated P2RX7B receptor in immune cells [23]. Accordingly, we also show that ATP did not enhance TO-PRO™-3 fluorescence in non-immune cells. Lung CD45^−^ cells comprise endothelial cells, fibroblasts, and alveolar epithelial cells of type I (AEC I) and type II (AEC II). They all express P2RX7 except for AEC II. It has been shown in vitro and ex vivo studies that they express a functional receptor (macropore opening, calcium influx, IL-1β release) [24, 25] but we do not seem to detect an activation *in vivo* with our protocol by measuring TO-PRO™-3 fluorescence in CD45^−^ cells. This could be due to the lower levels of expression of P2RX7 in CD45^−^ cells than in CD45^+^ cells (Fig. 3B, supp Fig. 2B) and the heterogeneity of the amplitude of its activation in these cells that is lost when looking at overall CD45^−^ cells. One possible way to overcome this is to analyze TO-PRO™-3 fluorescence in each CD45^−^ cell type by including specific markers. However, we could not exclude that the overall macropore activity of P2RX7 is impaired by the expression of truncated splice variants as previously described in mouse astrocytes [26]. Surprisingly, we observed that TO-PRO™-3 fluorescence was decreased in P2RX7^+^CD45^−^ cells in ATP-mice (Fig. 3D). It should be noted that the intensity of TOPRO™-3 fluorescence in P2RX7-expressing CD45^−^ cells (Fig. 3D) is identical to the background value observed in *p2rx7*^*−/−*^ mice (Fig. 2C) accompanied by unchanged P2RX7 expression (Fig. 3B, supp Fig. 2B). Thus, these observations support the background signal of TO-PRO-3 fluorescence rather than an ATP-related effect.

We have also shown that the expression of P2RX7 was decreased in WT-ATP mice. P2RX7 activation by ATP stimulates membrane internalization [27] that results to the loss of expression of many surface proteins [28, 29] including P2RX7 [13, 14]. Indeed, prolonged stimulation with high levels of ATP results in decreased surface expression and internalization of the receptor in RAW cells [14], Caski cells and HEK 293 transfected with the human P2RX7 [13], at least 15 to 30 minutes after its activation depending on the cell type. Decreased expression of the receptor was also reported on human and mouse monocytes after sustained stimulation with bzATP [30]. Moreover, it has been shown that sustained activation of P2RX7 induces its cleavage at the plasma membrane by MMP-2, starting 15 minutes after its activation [15]. Altogether, these observations support the decrease of P2RX7 surface expression in lungs of mice due to high levels of ATP administration and duration of P2RX7 activation in this study.

Even though this protocol successfully detects P2RX7 activation in the lungs, it can be adapted for studying its activation in other organs by giving TO-PRO™-3 to the mice by intravenous (i.v.) route. YO-PRO-1 (an analog of TO-PRO™-3) and propidium iodide (PI) are also used to study macropore formation. They have been given i.v. in mice and rats to study *in vivo* cell death in lungs [31, 32], brain [33, 34] and liver [35]. However, TO-PRO™-3’s time to access and penetrate the organ of interest should be carefully assessed in order to study the activation of P2RX7.

To go further, this protocol could also be helpful to determine the *in vivo* efficacy of compounds targeting P2RX7, to potentially adapt their administration route or their dosage or even to identify the targeted cell type of the compounds. HEI3090 is a novel positive modulator of P2RX7 that enhances calcium influx, macropore opening and IL-18 release, as determined by in vitro and ex vivo experiments. Although we showed that HEI3090 enhances IL-18 release *in vivo* [5], it is of interest to study its impact on P2RX7 activation *in vivo*, especially since HEI3090 is a promising molecule for treatment of lung cancer [5].

## Conclusions

We described a protocol assessing P2RX7 activation *in vivo*, by taking advantage of its unique property of macropore formation. Given the immunomodulatory functions of P2RX7 and the increasing number of molecules aiming to modulate its activity, this protocol represents an important advance in the field of purinergic signaling.

## Methods

The described methods include the description of the work, along with general guidelines that can be used for implementing the strategy with various compounds described to modulate P2RX7 activity.

### Animals

Eight weeks old WT C57BL/6 J Olahsd mice are from Envigo, (Gannat, France), and 8 weeks old *p2rx7*^*−/−*^ (B6.129P2-P2rx7tm1Gab/J) backcrossed more than 10 times with WT C57BL/6 J Olahsd and are inbreeded in our animal facility. Mice were housed under standardized light–dark cycles in a temperature-controlled air-conditioned environment under specific pathogen-free conditions at IRCAN, Nice, France, with free access to food and water. All experiments were approved by the committee for Research and Ethics of the local authorities (CIEPAL #772, protocol number MESRI APAFIS #33150–2021091413316813) and followed the European directive 2010/63/UE, in agreement with the ARRIVE guidelines. Experiments were performed in accord with animal protection representative at IRCAN.

**Fig. 4:**
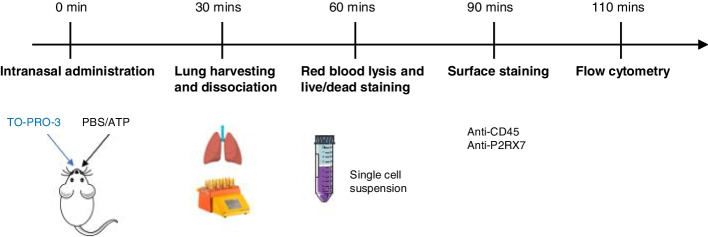
Schematic protocol assessing P2RX7 activity *in vivo*

Protocol description is summarized in Fig. 4. Measuring P2RX7 activity *in vivo* requires 5 sequential and mandatory steps as illustrated below.

#### Time 0 - Intranasal Administration

1. Anesthetize mouse i.p. with a mixture of 25 mg/kg ketamine and 2.5 mg/kg xylazine using 1 ml syringe with a 26G needle.
2. Place mouse in a box with 5% isoflurane for 1 minute.
3. When asleep, mouse is held upright and was given 25 μl of TO-PRO™-3 (1 μM/kg) in one nostril then 25 μl of ATP (100 mM) or 1X PBS in another using a micropipette.

*/!\ Intranasal administration should be slow and should follow the respiration rate of the mouse.*

*/!\ Mice should be anesthetized and processed for intranasal delivery one by one.*

*/!\ As soon as the 25 μl + 25 μl are completely taken up by the mouse, start the 30 minutes timer.*

#### Time 30 Minutes – Mouse Sacrifice and Lung Harvesting

1. Sacrifice mouse by cervical elongation
2. Spray mouse thorax with 70% ethanol. Harvest lungs using fine and sharp scissors and pliers.
3. Separate the lobes. Transfer the lobes into a GentleMACS™ C-tube (Miltenyi Biotech) containing the enzymatic buffer for dissociation (Lung dissociation kit, Miltenyi Biotech)
4. Dissociate lungs using the 37_m_LDK1 program on a GentleMACS™ Octo dissociator with heaters (Miltenyi Biotech)
5. At the end of the program, resuspend cells and pass them through a 100 μm cell strainer on top of a 50 ml tube for single cell suspensions. Wash the GentleMACS™ C-tube with 1X Buffer S (Lung dissociation kit, Miltenyi Biotech) for maximum retrieval of cells and pass them though the 100 μm cell strainer.
6. Discard the cell strainer and centrifuge the tube at 1200 rpm 4 °C for 5 minutes.

*/!\ Keep cells in the dark at all times, and on ice except during the dissociation program.*

#### Time 60 Minutes – Red Blood Lysis and Live/Dead Staining

1. Aspirate completely the supernatant and resuspend pellet gently with 1 ml of ice-cold ACK lysis buffer (Gibco) for 30 seconds to lyse red blood cells.
2. Add 30 ml of 1X PBS for stopping the reaction.
3. Centrifuge at 1200 rpm 4 °C for 5 minutes
4. Count cells
5. Add 1 million of lung single cells in 150 μl 1X PBS in a 5 ml polypropylene tube
6. Add 150 μl of Green Live/Dead stain (2X) (Invitrogen) or 1X PBS (unstained control) into the 5 ml polypropylene tube

*/!\ Prepare appropriate number of samples without Live/Dead staining for single-stained controls*

7. Vortex and incubate in the dark at room temperature for 30 minutes
8. Add 3 ml of FACS buffer (PBS 5% FBS 0.5% EDTA) into the 5 ml polypropylene tube
9. Centrifuge at 1200 rpm 4 °C for 5 minutes
10. Aspirate supernatant completely and resuspend cells in 100 μl FACS Buffer

*/!\ Keep cells in the dark at all times, and on ice unless otherwise stated.*

#### Time 90 Minutes – Surface Staining

1. Transfer cell suspension to a 96 well V plate
2. Centrifuge plate at 1300 rpm 4 °C for 3 minutes
3. Flick the plate
4. Block Fc receptors using anti-CD16/32 (dil 1/100, BD Biosciences) diluted in FACS Buffer (1X PBS, 5% FBS, 0.5% EDTA) in the dark under agitation for 10 minutes
5. Centrifuge plate at 1300 rpm 4 °C for 3 minutes
6. Flick plate
7. Add antibody mix diluted in FACS buffer and homogenize immediately using a multichannel pipette.
  Antibodies: CD45 BUV395 (dil 1/100, BD Biosciences), P2RX7 PE (dil 1/8, Biolegend) or isotype control rat IgG2b κ PE (dil 1/8, Biolegend).

*/!\ Prepare appropriate single-stained controls*

8. Incubate at 4 °C in the dark under agitation for 20 minutes
9. Add 100 μl of FACS buffer per well using a multichannel pipette
10. Centrifuge plate at 1300 rpm 4 °C for 3 minutes
11. Flick the plate
12. Resuspend cell in 100 μl of FACS buffer

*/!\ Keep cells in the dark at all times, and on ice.*

#### Time 110 Minutes – Flow Cytometry Analysis

1. Cells were acquired using the CytoFLEX LX (Beckman Coulter)
2. Samples were analyzed using FlowJo (LLC)

#### Materials

1. 1 ml syringe omnifix-F (Braun, catalog number 9161406 V)
2. 26G needle (BD Microlance 3, catalog number 304300)
3. Pipette
4. Pipette tips
5. Fine scissors
6. Sharp scissors
7. Pliers
8. GentleMACS™ C-tube (Miltenyi Biotech, catalog number 130–093-237)
9. 100 μm cell strainer (Falcon, catalog number 352360)
10. Polypropylene 50 ml tube (Falcon, catalog number 352070)
11. Polypropylene 5 ml tube (Falcon, catalog number 352096)
12. 96-well clear V bottom plates (Greiner, catalog number 651101)
13. Serological pipette (Falcon, catalog number 357543)
14. Serological pipette gun
15. Multi-channel pipette (Starlab, catalog number 57112–330)

#### Reagents

1. Ketamine (Virbac, catalog number 03597132111010)
2. Xylazine (Sedaxylan, Dechra, catalog number 08714225151523)
3. Isofluorane / Aerrane (Baxter, catalog number DAGG9223)
4. TO-PRO™-3 (Life Technologies, catalog number T3605)
5. ATP (Sigma-Aldrich, catalog number A6419)
6. 1X Dulbecco’s PBS (Gibco, catalog number 14190144)
7. 70% ethanol
8. Double distilled water
9. 20X Buffer S, enzyme D and enzyme A from the mouse lung dissociation kit (Miltenyi Biotech, catalog number 130–095-927)
10. ACK lysis buffer (Gibco, catalog number A1049201)
11. LIVE/DEAD™ fixable green dead cell staining kit (Invitrogen, catalog number L23101)
12. Fetal bovine serum (FBS)
13. Ethylenediaminetetraacetic acid (EDTA) (Invitrogen, catalog number 15575020)
14. CD16/32 (BD Biosciences, catalog number 553141)
15. CD45 BUV395 (BD Biosciences, catalog number 564279)
16. P2RX7 PE (Biolegend, catalog number 148703)
17. Rat IgG2b κ PE (Biolegend, catalog number 400607)

#### Equipment

1. Anesthesia machine and box (Anesteo)
2. GentleMACS™ Octo dissociator with heaters (Miltenyi Biotech, catalog number 130–096-427)
3. Tube and plate centrifuge (Thermofischer scientific, catalog number 75009527)
4. Plate agitator
5. CytoFLEX LX (Beckman Coulter)

#### Reagent Preparation

*/!\ Prepare all reagents in sterile conditions using a PSM II tissue culture hood*

1. Anesthetics
  Prepare 25 mg/kg of ketamine and 2.5 mg/kg of xylazine in 1X Dulbecco’s PBS as per 250 μl of mix per 25 g of mouse.
2. Lung dissociation buffer
  Dilute 20X Buffer S in double distilled water and store at 4 °C. Resuspend enzyme A and enzyme D with 1X Buffer S according to manufacturer’s instructions. Add 2.4 ml of 1X Buffer S, 15 μl of enzyme A and 100 μl of enzyme D per GentleMACS™ C-tube (Miltenyi Biotech)
3. ATP
  Prepare 100 mM stock of ATP in sterile 1X PBS, pH 6.8, aliquot and store at − 80 °C.

*/!\ Avoid freeze/thaw cycles*

4. TO-PRO™-3

Dilute TO-PRO™-3 in 1X PBS to 1 μM/kg.

*/!\ Manipulate TO-PRO™-3 in the dark.*

*/!\ Aliquot TO-PRO™- 3 stock and avoid freeze/thaw cycles*

5. LIVE/DEAD™ fixable green dead cell staining kit

Dilute LIVE/DEAD™ in 1X PBS to 2X working concentration per tube. Add 0.4 μL LIVE/DEAD™ in 150 μL per tube.

*/!\ Manipulate LIVE/DEAD™ green in the dark*

6. /!\ *Prepare mix of appropriate volume*
7. FACS Buffer
  Prepare 500 ml of FACS Buffer containing 1X Dulbecco’s PBS, 5% FBS, 0.5% EDTA and store at 4 °C.

*/!\ Keep on ice at all times*

8. Antibody mix cocktail
  Dilute CD45 BUV395 (dil 1/100) and P2RX7 PE (dil 1/8) or isotype control rat IgG2b κ PE (dil 1/8) in FACS Buffer.

*/!\ Keep on ice at all times and manipulate in the dark.*

#### Software

1. CytExpert (Beckman Coulter)
2. FlowJo (LLC) or any other flow cytometry software
3. Graphpad prism

#### Statistical Analyses

All analyses were carried out using Prism software (GraphPad). Mouse experiments were performed on at least *n* = 5 individuals, as indicated. Mice were equally divided for treatments and controls. Data is represented as mean values and error bars represent SEM. Two-tailed Mann–Whitney and unpaired *t*-test were used to evaluate the statistical significance between groups.

## Supplementary Information

**Additional file 1: Supp Fig. 1.** ATP administration does not affect the percentage of TO-PRO™-3^+^ cells. **Supp Fig. 2.** P2RX7 fluorescence in lung cells of WT and p2rx7^−/−^ mice.

## Data Availability

All data generated and analyzed during this study are included in this article and its supplementary files.
