# Supplementary material for: Protocol for Evaluating In Vivo the Activation of the P2RX7 Immunomodulator
Source: Biol Proced Online. 2023 Jan 4;25:1. doi: 10.1186/s12575-022-00188-6 (PMC9811721; doi:10.1186/s12575-022-00188-6)
Supplement: Supplementary file 1 — Additional file 1: Supp Fig. 1. ATP administration does not affect the percentage of TO-PRO™-3+ cells. Supp Fig. 2. P2RX7 fluorescence in lung cells of WT and p2rx7−/− mice. [file 12575_2022_188_MOESM1_ESM.docx]

**Supplementary materials**

**A**

**B**

**C**

Whole lung

CD45^+^

CD45^-^

**D**

**E**

**F**

**Supp Fig. 1.** ATP administration does not affect the percentage of TO-PRO^TM^-3^+^ cells

1. Percentage of TO-PRO^TM^-3^+^ cells in whole lung of WT and *p2rx7^-/-^* mice.
2. Percentage of TO-PRO^TM^-3^+^ cells in CD45^+^ cells and **C.** CD45^-^ cells in WT and *p2rx7^-/-^* mice
3. Percentage of TO-PRO^TM^-3^+^ cells among P2RX7^+^ cells in WT mice
4. Percentage of TO-PRO^TM^-3^+^ cells among P2RX7^+^CD45^+^ cells in WT mice
5. Percentage of TO-PRO^TM^-3^+^ cells among P2RX7^+^CD45^-^ cells in WT mice

Whole lung

CD45^+^

CD45^-^

**A**

**B**

**Supp Fig 2.** P2RX7 fluorescence in lung cells of WT and p2rx7^-/-^ mice

1. GMFI of P2RX7^+^ in WT mice
2. GMFI of P2RX7^+^ in WT mice in CD45^-^ or CD45^+^ cells in WT and *p2rx7^-/-^* mice
